# Regulated Proteolytic Processing of Reelin through Interplay of Tissue Plasminogen Activator (tPA), ADAMTS-4, ADAMTS-5, and Their Modulators

**DOI:** 10.1371/journal.pone.0047793

**Published:** 2012-10-17

**Authors:** Dimitrije Krstic, Myriam Rodriguez, Irene Knuesel

**Affiliations:** Institute of Pharmacology and Toxicology, University of Zurich, Zurich, Switzerland; University of Kentucky, United States of America

## Abstract

The extracellular signaling protein Reelin, indispensable for proper neuronal migration and cortical layering during development, is also expressed in the adult brain where it modulates synaptic functions. It has been shown that proteolytic processing of Reelin decreases its signaling activity and promotes Reelin aggregation *in vitro*, and that proteolytic processing is affected in various neurological disorders, including Alzheimer's disease (AD). However, neither the pathophysiological significance of dysregulated Reelin cleavage, nor the involved proteases and their modulators are known. Here we identified the serine protease tissue plasminogen activator (tPA) and two matrix metalloproteinases, ADAMTS-4 and ADAMTS-5, as Reelin cleaving enzymes. Moreover, we assessed the influence of several endogenous protease inhibitors, including tissue inhibitors of metalloproteinases (TIMPs), α-2-Macroglobulin, and multiple serpins, as well as matrix metalloproteinase 9 (MMP-9) on Reelin cleavage, and described their complex interplay in the regulation of this process. Finally, we could demonstrate that in the murine hippocampus, the expression levels and localization of Reelin proteases largely overlap with that of Reelin. While this pattern remained stable during normal aging, changes in their protein levels coincided with accelerated Reelin aggregation in a mouse model of AD.

## Introduction

Reelin, a highly conserved extracellular signaling protein that played a crucial role in the evolution of the cerebral cortex in mammals [1], [2] is not only essential for proper neurodevelopment [3], [4], but is also expressed in the adult brain where it modulates synaptic plasticity and thus is necessary for neuronal functions involved in learning and memory [5], [6]. In addition, in several brain disorders, including AD, Reelin protein levels, its post-translational modifications, and Reelin proteolytic processing are found to be dysregulated [7]–[11].

Reelin exerts its functions by binding to apolipoprotein E receptor 2 (ApoER2) and very low density lipoprotein receptor (VLDLR) [12], [13], thereby inducing their clustering [14] and subsequent phosphorylation of the adaptor protein Disabled-1 (Dab-1) [15]. This process activates cytosolic kinase pathways involving phosphatidylinositol 3-kinase (PI3K), and protein kinase B (Akt/PKB) [15], [16] leading to the inhibition of glycogen synthase kinase 3β (GSK3β) [16] and suppression of Tau hyperphosphorylation [17].

**Figure 1:**
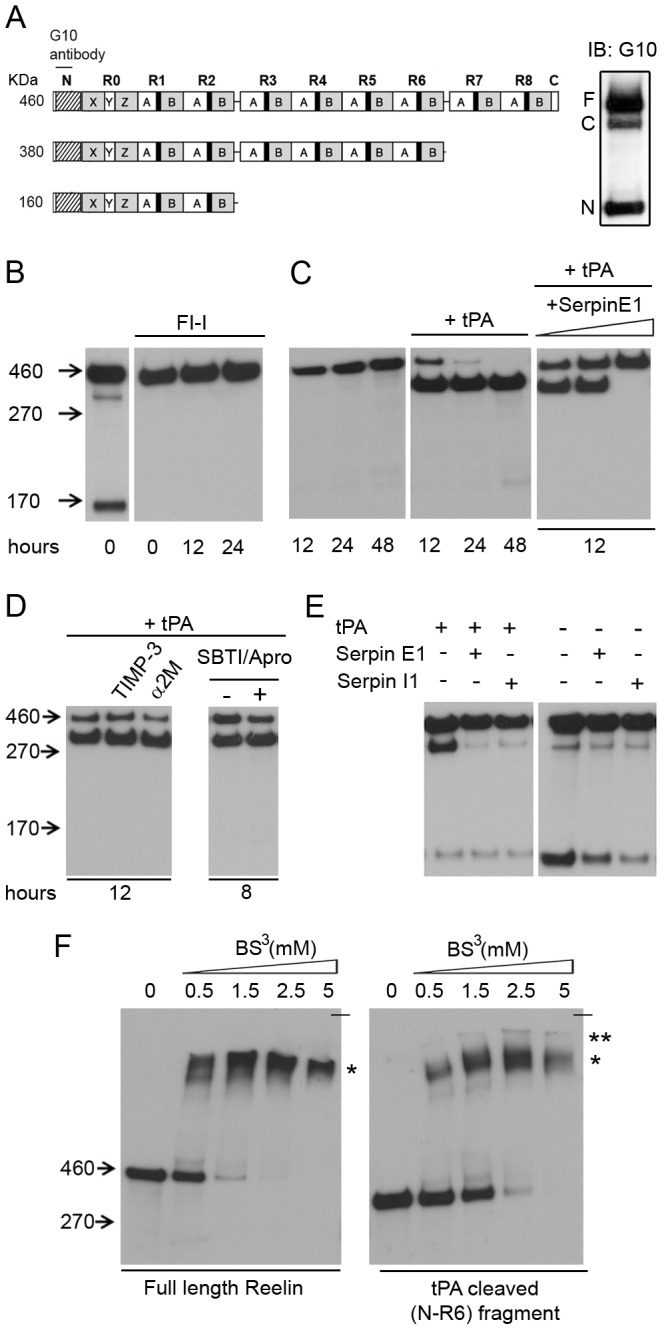
tPA cleaves Reelin at its C-terminal cleavage site. (**A**) Schematic representation of the Reelin fragments detected with G10 anti-Reelin antibody (left) and immunoblot (IB) of hippocampus lysate from a 3 month-old wild-type mouse, showing full-length Reelin (F, 460 kDa), C-terminal cleaved (C, 380 kDa), and N-terminal cleaved (N, 160 kDa) Reelin fragments (right). (**B-F**) Anti-Reelin IB (G10, N-terminal antibody). (**B**) 100 mM Furin Inhibitor I (FI-I) inhibits Reelin cleavage in HeLa cells expressing Reelin. After 12 hours incubation of FI-I or DMSO alone, medium was collected (0 h) and further incubated at 37°C to check for potential Reelin degradation. (**C**) Recombinant tPA (25 ng/µl) cleaves Reelin at its C-terminal cleavage site. Recombinant serpin E1 (12.5, 37.5, 100 ng/µl) inhibits tPA action. (**D**) Neither the metalloproteinase inhibitors TIMP-3 (40 ng/µl) or α2M (40 ng/µl), nor the trypsin inhibitors SBTI/Aprotinin (1000 and 300 ng/µl) affect Reelin cleavage by tPA (25 ng/µl). (**E**) Co-expression of tPA and its inhibitors in Reelin expressing HEK293 cells. Sample (medium) for IB was collected after 12 h incubation. (**F**) Cross-linking Reelin with BS3 (30 minutes on ice) revealed that C-terminal cleavage does not affect Reelin dimerization (asterisk). The additional band observed with C-terminally cleaved Reelin (two asterisks) may represent higher-order oligomers. Note that the top of the gel is marked with the horizontal line. Indicated hours represent incubation times. All IB blots are representatives of three independent experiments.

Reelin itself is subjected to proteolytic processing at two main sites (Fig. 1A) [3], [18]. Although the physiological function of this proteolytic fragmentation is still not fully understood, current data suggests that the N-terminal Reelin region (N-R2) is required for protein homodimerization and signaling [19], [20], while the central region (R3-6) that represents a minimal binding unit for ApoER2 and VLDLR [21], [22] is involved in Reelin oligomerization [23], and interaction with the amyloid precursor protein (APP) [24], [25]. The C-terminal fragment (R7-C) is required for proper protein folding [26] and full signaling activity [27], [28]. In addition, it has been demonstrated *in vitro* that the Reelin N-terminal fragment is prone to aggregate [29] and that Reelin lacking the N-terminal domain can also form protein complexes that are larger than functional dimers [20]. However, these complexes may not be stable without the aggregation-prone N-terminal domains [29]. These observations have been recently complemented by *in vivo* findings showing that Reelin accumulates in amyloid-like aggregates during the course of aging [30]. Moreover, formation of these aggregates is accelerated both after prenatal immune challenge and in transgenic AD mice [30], suggesting that changes in Reelin proteolytic processing or degradation during pathological aging may be responsible for its accelerated aggregation. However, despite an obvious modulatory effect of Reelin proteolytic processing on its signaling activity and the described dysregulation of Reelin processing in several neurological disorders [31], [32], the proteases (peptidases) responsible for Reelin processing remained unidentified.

Here we demonstrate that the serine protease tissue plasminogen activator (tPA) cleaves Reelin at its C-terminal site, and that the extracellular matrix (ECM) metalloproteinase ADAMTS-4 (aggrecanase-1) is able to cleave Reelin at both the C- and N-terminal site. Moreover, we found that ADAMTS-5 (aggrecanase-2) is not only able to cleave Reelin at both cleavage sites, but also engaged to further degrade the Reelin N-terminal domain. Importantly, we show that Reelin and its proteases are co-expressed in the hippocampus of wild-type animals. Although no differences in the levels of Reelin cleavage and Reelin proteases were detected between young (4 weeks) and old (16 months) wild-type mice, we observed a significant accumulation of smaller N-terminal Reelin fragments, potentially related to reduced degradation/clearance in older animals. Finally, immunohistochemical analyses revealed significant differences in the levels of Reelin proteases in old (15 months) 3xTg-AD mice as compared to age-machted control animals [33], coinciding with accelerated Reelin aggregation *in vivo*.

## Results

### Screening Strategy

To screen for Reelin proteases, we took advantage of the observation that P19 mouse embryonic carcinoma cells produce and secrete Reelin, but only upon retinoic acid-induced differentiation into neurons, they start to cleave the protein [34]. Hence, we scrutinized previously published microarray data [35] for proteases whose expression was upregulated upon differentiation of P19 cells. We focused on matrix metalloproteinases and extracellular serine proteases, since previous work suggested members of these families to be involved in Reelin processing [18], [34]. Consulting the GeneCards encyclopedia (www.genecards.org) and MEROPS peptidase database (http://merops.sanger.ac.uk), we identified 19 serine and 20 metalloproteinases being significantly upregulated (Table S1). Furthermore, to obtain uncleaved Reelin for an *in vitro* protease screen, we modified a recently published protocol [19] (see Materials and Methods) that allowed us to produce full-length uncleaved Reelin (FL-Reelin), which was stable at 37°C for at least 48 h (Fig. 1B).

### Tissue Plasminogen Activator (tPA)

Among 19 identified serine proteases (Table S1), tPA was of particular interest to us since it was previously claimed that tPA similarly to urinary-type plasminogen activator (uPA) can degrade Reelin in plasma [36]. Hence, we incubated activated recombinant tPA with FL-Reelin and checked for the presence of cleaved fragments. Indeed, tPA cleaved FL-Reelin, however unlike uPA, specifically at the C-terminal cleavage site (Fig. 1C). Newly formed N-terminal-containing Reelin fragments were not subjected to further degradation by tPA, even after 48 h at 37°C (Fig. 1C). While this cleavage could be completely inhibited by the addition of the endogenous tPA inhibitor serpin E1 (Fig. 1C), neither matalloproteinase inhibitors (TIMP-3 and α-2-Macroglobulin - α2M) nor trypsin inhibitors (SBTI and Apro) could inhibit the tPA-mediated cleavage (Fig. 1D). To test if this finding could be replicated in cell cultures, we co-transfected HEK293 cells with two cDNA contructs encoding Reelin and tPA. In comparison to cells expressing Reelin only, cultures expressing also tPA showed increased C-terminal processing of secreted Reelin (Fig. 1E). Co-expression of serpin E1 (nexin) or I1 (neuroserpin) inhibited tPA protease activity, but rather unexpectedly also affected tPA independent N-terminal cleavage (Fig. 1E).

Since it has been shown that the Reelin N-terminal fragment (NR-2) is prone to aggregation [29] and that Reelin lacking its N-terminal fragment forms protein complexes larger than functional dimers [20], we tested if the C-terminal cleavage could similarly destabilize Reelin homodimer formation. Therefore, we covalently linked either FL- or C-cleaved Reelin with BS3 crosslinker [20] and then performed immunoblotting. While FL-Reelin was involved in the formation of the expected homodimers (asterisk, Fig. 1F), the C-cleaved Reelin fragments produced an additional immunoreactive Reelin band that ran slightly higher than the Reelin homodimers (two asterisks, Fig. 1F).

### ADAMTS-4

**Figure 2:**
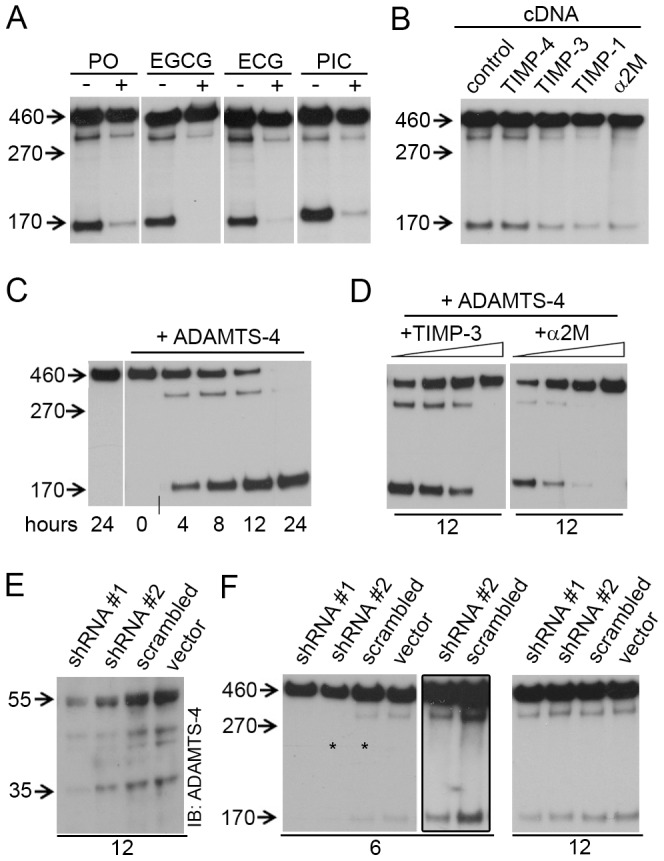
ADAMTS-4 cleaves Reelin at both the N- and C-terminus. (**A–D, F**) Anti-Reelin (G10, N-terminal antibody) immunoblots (IB). (**A,B**) Samples from Reelin-expressing HEK293 cells were collected after 12 hours incubation. (**A**) Metal chelator (PO, 50 µM), catechins (EGCG, 150 µM; ECG, 150 µM), and picetanol (PIC, 60 µM) inhibit Reelin cleavage. (**B**) Expression of TIMP-3, -1, and α2M, but not TIMP-4, inhibit Reelin cleavage. (**C**) Recombinant ADAMTS-4 (10 ng/µl) processes Reelin at both cleavage sites. Short vertical line at the bottom of the blot denotes that the last lanes, from the same blot, were joined for visual presentation. (**D**) Recombinant TIMP-3 (1.5, 3, 6, 12 ng/µl) and α2M (44, 72, 88,100 ng/µl) inhibit the proteolytic action of ADAMTS-4 on full-length Reelin. (**E**) Anti-ADAMTS-4 immunoblots (IB) showing reduction of ADAMTS-4 protein levels in HeLa cells transfected with two different ADAMTS-4 shRNA constructs, but not with scrambled shRNA nor with empty vector. (**F**) ADAMTS-4 shRNA transiently inhibited Reelin cleavage in Reelin-expressing HeLa cells. The frame emcompasses overexposed lanes from the blot on the right (asterisks) for better visualization of the Reelin fragments.

Previous work suggested member(s) of the adamalysin or astacin families of metalloproteinases being involved in Reelin cleavage [18], [19]. In addition, it was shown that a Reelin protease is secreted into the extracellular matrix that has a high affinity to heparin [19]. Out of the 20 metalloproteases upregulated after P19 cell differentiation (Table S1) seven belonged to the ADAMTS (a disintegrin and metalloproteinase with thrombo-spondin motifs) family, a group of secreted proteases with high heparin-affinity [37]. Hence, to test if members of this family are involved in Reelin processing, we added the catechins gallate esters Epigallocatechin gallate (EGCG) and Epicatechingallate (ECG), known to inhibit the activity of the ADAMTS family [38], to HEK293 cells expressing Reelin. The addition of the metal chelator 1,10-Phenanthroline (PO) served as positive control [18]. If applied at high concentrations, EGCG and ECG inhibited both the C- and N-terminal Reelin cleavage (Fig. 2A), however, lower concentrations had a more pronounced effect on the N-terminal cleavage (Fig. S1A,B).

Among the ADAMTS members, ADAMTS-4 (aggrecanase-1) was of a particular interest to us, since it is a known inflammation-inducible protease [39], [40] and immune-challenged wild-type mice show a pronounced acceleration in Reelin aggregation [30], presumably as a consequence of increased Reelin cleavage at its N-terminus [20], [29] or C-terminal site (Fig. 1F). Thus, adding piceatannol (PIC), a selective inhibitor of ADAMTS-4 and -5 [41], to the cell cultures induced a strong inhibition of Reelin cleavage at both, the N- and C-terminal site (Fig. 2A and Fig. S1C). In addition, co-expression of Reelin with the endogenous ADAMTS-4 inhibitors TIMP-1 or TIMP-3 [42], but not with TIMP-4, blocked Reelin proteolytic processing in the HEK293 cell medium (Fig. 2B). The same effect was observed by co-expression of Reelin with α-2-Macroglobulin (α2M), shown to potently inhibit ADAMTS-4/-5 [43]. Next, we incubated recombinant active ADAMTS-4 with FL-Reelin and demonstrated efficient cleavage at both, the C- and N-terminal cleavage site (Fig. 2C). This processing could be completely inhibited by recombinant TIMP-3 or α2M (Fig. 2D), but not by trypsin inhibitors (Fig. S1D).

To silence ADAMTS-4 expression *in vitro,* we co-transfected HeLa cells with Reelin and shRNA against ADAMTS-4. We obtained a shRNA transfection rate of 80%, as estimated by the numerical density of GFP-positive cells, and achieved a 50-70% reduction in ADAMTS-4 protein levels 24 h post-transfection (Fig. 2E). After medium exchange to serum free Aim-V, cells were left for additional 6-12 h in the incubator at 37°C. Interestingly, shRNA-mediated knock-down of ADAMTS-4 and concomitant inhibition of Reelin cleavage in the medium was confirmed after 6 h, but not after 12 hours (Fig 2F). Similar experiments involving inhibition of tPA activity with a shRNA approach, did not lead to a decrease in Reelin cleavage in HeLa cells (data not shown).

### MMP-9

**Figure 3:**
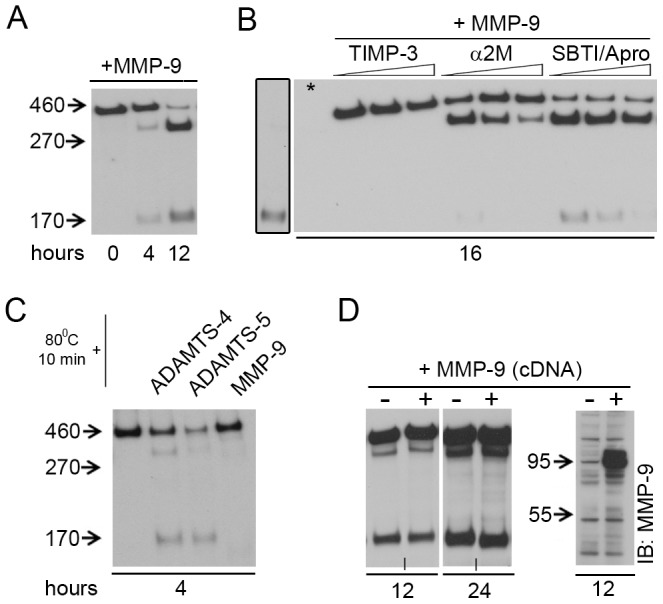
MMP-9 activates Reelin-cleaving proteases. (**A–D**) Anti-Reelin (G10, N-terminal antibody) immunoblots (IB). (**A**) Recombinant MMP-9 (10 ng/µl) induces Reelin cleavage. (**B**) Action of MMP-9 (20 ng/µl) on Reelin cleavage could be suppressed with TIMP-3 (10, 15, 20 ng/µl), α2M (10, 20, 40 ng/µl), and also with trypsin inhibitors SBTI and Apro (500 and 150, 1000 and 300, 2000 and 600 ng/µl). The framed lane belongs to the same IB (asterisk on the right blot), but was overexposed for visualization of cleaved Reelin. (**C**) Incubation of FL-Reelin and recombinant ADAMTS-4 (10 ng/µl), ADAMTS-5 (20 ng/µl), or MMP-9 (10 ng/µl) after heating the FL-Reelin medium at 80°C for 10 min. (**D**) Expression of the MMP-9 cDNA did not increase Reelin cleavage in Reelin expressing HEK293 cells (left), although we could confirm the synthesis of MMP-9 in these cells using anti-MMP-9 antibody (right). Short vertical lines at the bottom of the blots denote that the last lanes, from the same blot, were joined for visual presentation.

Under physiological conditions, proADAMTS-4 is activated either by Furin or matrix metalloproteinase 9 (MMP-9 or gelatinase B) [44]. Since Furin inhibitors block the maturation of ADAMTS-4 [45] and also inhibit Reelin processing [19], we wondered if addition of proteolytically active MMP-9 into FL-Reelin medium could result in ADAMTS-4 activation and subsequent induction of Reelin processing. Indeed, the addition of MMP-9 to FL-Reelin medium induced efficient cleavage of FL-Reelin (Fig. 3A). While MMP-9-mediated Reelin processing could be fully inhibited at both sites by the addition of TIMP-3, the addition of α2M, a potent inhibitor of ADAMTS-4 and -5, had a more pronounced effect on the N-terminal cleavage (Fig 3B). Interestingly, also the trypsin protease inhibitors (SBTI/Apro) suppressed the N-terminal Reelin cleavage, however, did not affect the processing at the C-terminal site (Fig. 3B). To check if MMP-9 itself is able to cleave Reelin, we incubated FL-Reelin medium at 80°C for 10 minutes, and subsequently incubated it on ice for 5 minutes, a procedure that resulted in the denaturation of proteases present in the medium, but neither affected FL-Reelin stability (Fig. S1E) nor the confirmation required for proteolytic processing (Fig. 3C). While the addition of recombinant ADAMTS-4 resulted in the generation of the C- and N-terminally cleaved Reelin fragments, the addition of functionally active MMP-9 into heat-treated medium did not result in Reelin cleavage (Fig. 3C). In line with this observation, co-expression of Reelin and MMP-9 in HEK293 cells did not increase Reelin proteolytic processing (Fig. 3D).

### ADAMTS-5

**Figure 4:**
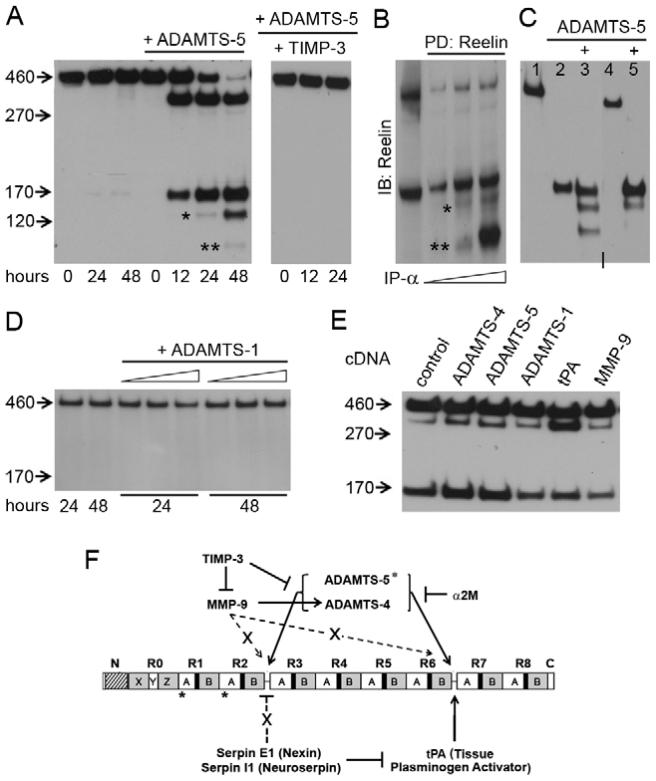
ADAMTS-5 degrades Reelin. (**A–D**) Anti-Reelin (G10, N-terminal antibody) immunoblots (IB). (**A**) Recombinant ADAMTS-5 (40 ng/µl) cleaves Reelin at both, its N- and C-cleavage site and further degrades the N-terminal fragment (asterisks). ADAMTS-5 activity is abolished after addition of TIMP-3 (20 ng/µl). (**B**) Pull-down (PD, using the G10 anti-Reelin antibody, diluted at 1:200, 1:100, 1:50) and subsequent IB with the same antibody revealed the existence of smaller N-terminal fragments (asterisks) in *wild-type* hippocampus homogenates. (**C**) Full length Reelin after 52 h incubation at 37°C (lane 1). ADAMTS-5 (80 ng/µl) was added to the FL-Reelin medium (indicated by +), which was previously incubated for 48 h with ADAMTS-4 (20 ng/µl, lane 2) or tPA (50 ng/µl, lane 4).Cleaved Reelin was incubated with ADAMTS-5 for additional 4 hours at 37°C (lanes 3 and 5). (**D**) Recombinant ADAMTS-1 (10, 20, 40 ng/µl) does not cleave Reelin. (**E**) Reelin enriched-medium was transferred to HEK293 cells expressing the indicated protease. The samples were collected 24 h after the medium transfer. (**F**) Schematic summary of Reelin processing and the possible modulations of this process. Dashed lines symbolize indirect effects of serpins and MMP-9 on Reelin processing, which are not mediated by tPA and ADAMTS-4, respectively. X = unknown ECM mediator.

Since it was shown that ADAMTS-4 and ADAMTS-5 (aggrecanase-2) have a number of common substrates and overlapping physiological roles [46], and that both ADAMTS-4 and -5 are inhibited by α2M and piceatannol [41], [43], we tested if ADAMTS-5, that was also identified in our protease screen (Table S1), could cleave Reelin. Although shRNA-mediated knock-down of ADAMTS-5 in HEK293 cells did not affect Reelin cleavage in the medium (data not shown), incubation of FL-Reelin with recombinant active ADAMTS-5 led to formation of Reelin proteolytic fragments, a process that could be fully inhibited by the addition of TIMP-3 (Fig. 4A). However, in addition to the previously described Reelin fragments [3], [18], ADAMTS-5 produced also two additional fragments at approx. 130 and 100 kDa (asterisks, Fig. 4A). To check if these fragments were also present *in vivo*, we enriched the N-terminal containing Reelin fragments from hippocampus lysates by a pull-down assay using anti-Reelin N-terminal (G10) antibody. Subsequent immunoblotting revealed the presence of two additional Reelin fragments of approx. 130 and 100 kDa in the lysate (Fig. 4B), in line with the observation that the N-terminal domain is processed/degraded further into smaller fragments (Fig. S1F). Accordingly, incubation of stable Reelin N-terminal fragments (Fig. 4C, lane 2; obtained by incubation of FL-Reelin with ADAMTS-4) or of stable Reelin C-terminally cleaved fragments (Fig. 4C, lane 4; obtained by incubation of FL-Reelin with tPA) were degraded further after addition of ADAMTS-5 (Fig. 4C, lanes 3 and 5).

The fact that both ADAMTS-4 and -5 proteolytically process Reelin raised the question whether other members of the aggrecanase family [47] were also able to cleave Reelin. Hence, we incubated FL-Reelin with active ADAMTS-1 (aggrecanase-3), but found no evidence of proteolytic cleavage, neither at 24 h nor at 48 h of incubation (Fig. 4D). Overexpression of ADAMTS-1 in HEK293 cells did not influence Reelin cleavage either (Fig. S1H).

### Complex interplay of ECM proteases in Reelin processing

Based on previous data indicating that the cleavage of Reelin takes place post-secretion in the extracellular matrix [18], we wanted to rule out that co-expression of Reelin and its putative protease in the same cells may result in non-specific Reelin cleavage. Hence, we transfected HEK293 cells either with Reelin or with either of the putative Reelin proteases. Reelin-expressing HEK293 cells were incubated for 12 h at 37°C and the Reelin-enriched medium was then collected and transferred to HEK293 cells expressing either one of the putative Reelin proteases. After the medium transfer, cells were left in the incubator for additional 24 h. Subsequent analysis of the respective medium confirmed our findings that only tPA, ADAMTS-4, and ADAMTS-5 can process Reelin, while there was no change in Reelin processing in the presence of ADAMTS-1 or MMP-9, as compared to the control (Fig. 4E, see also schematic summary in Fig. 4F).

### Reelin proteases in the brain

To check for the protein levels and expression patterns of Reelin proteases in the brain, we performed biochemical analyses on brain lysates of 4 weeks and 16 months old wild-type animals, as well as immunohistochemical analyses involving perfusion-fixed brains of 15 months old AD-mice (3xTg-AD) and their age-matched non-transgenic controls [33].

**Figure 5:**
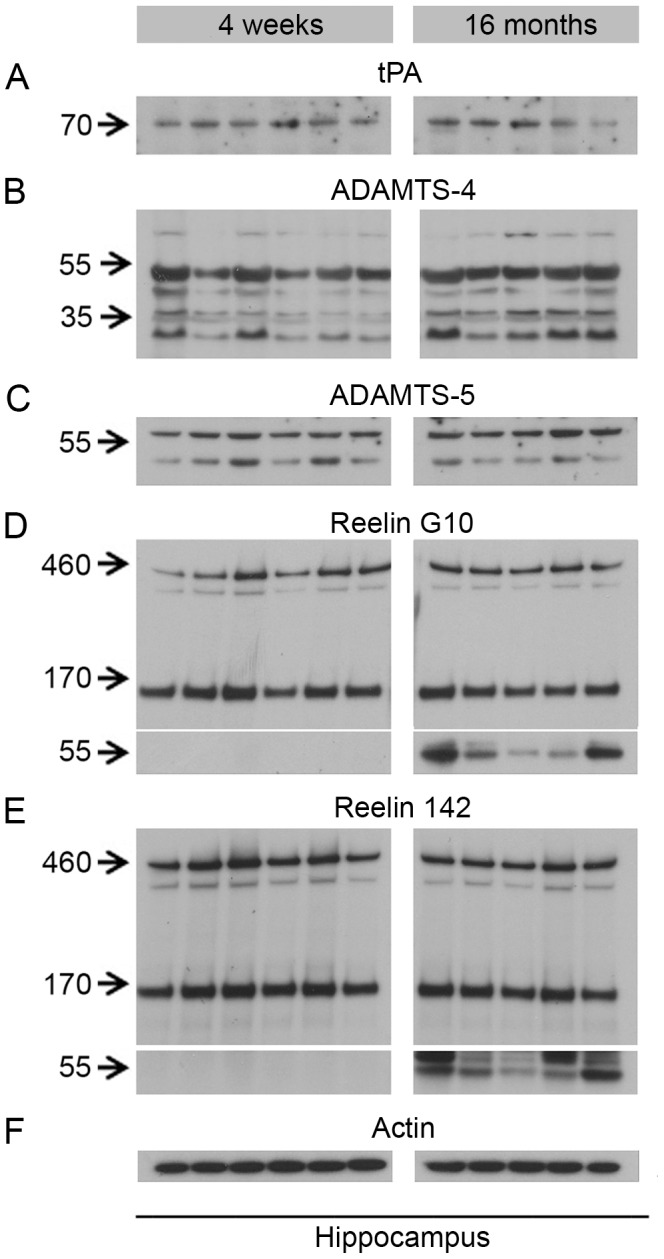
Biochemical analysis of Reelin preoteolytic processing and protein levels of the identified Reelin proteases in young and old wild-type mice. Immunoblots using (**A**) anti-tPA (H-90), (**B**) anti-ADAMTS-4 (PA1-1749A), (**C**) anti-ADAMTS-5 (ab41037), (**D**) anti-Reelin (G10), (**E**) anti-Reelin (142), (**F**) anti-Actin (MAB1522) antibody. Lanes represent different animals. Hippocampus lysates from young (4 weeks) and old (16 months) animals were processed on the same gel and membrane. No difference in Reelin cleavage or levels of Reelin proteases is observed between young and old animals. However, a prominent Reelin-positive band of ∼60 kDa was selectively observed in the aged animals.

Biochemical analysis revealed the presence of all identified proteases in the hippocampus of wild-type animals, however showed no significant differences in protein expression levels between two age groups (Fig. 5A-C). Note that the antibodies raised against ADAMTS-4 and -5 also detected the cleaved isoforms of these proteases (Fig. 5B,C). In accordance with the stable levels of Reelin proteases, we did not observe any changes in Reelin cleavage in aged as compared to young animals either (Fig. 5D,E). Interestingly, in aged wild-type mice we observed an increase of the Reelin N-terminus-containing band at approx. 60 kDa (Fig. 5D). Specificity of this band was confirmed by using two different Reelin N-terminal antibodies (Fig. 5D,E, Fig. S1F).

**Figure 6:**
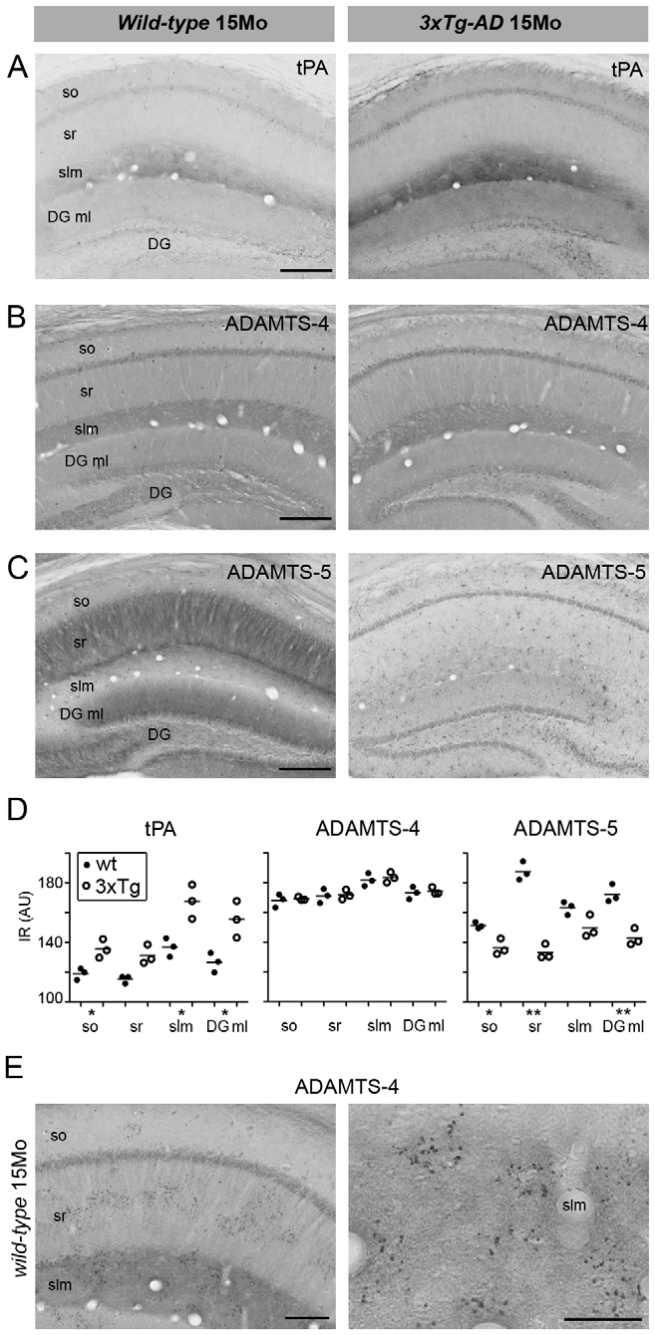
Immunohistochemical analysis of the localization of the Reelin proteases in the hippocampus of 3xTg-AD mice and their *non-transgenic* controls. (**A–C**) Immunoperoxidase labeling using anti-tPA (**A**), anti-ADAMTS-4 (**B**), and anti-ADAMTS-5 antibodies (**C**). (**D**) Semi-quantitative analysis of the tPA, ADAMTS-4 and -5 immunoreactivity (IR) in striatum oriens (so), striatum radiatum (sr), striatum lacunosum moleculare (slm), and Dentate Gyrus molecular layer (DG ml). AU, arbitrary units, represent mean background-corrected pixel brightness measured on 4 sections per animal (n = 3 per genotype). *p<0.05, **p<0.01, statistics based on unpaired *t-test* with Welch's correction. (**E**) Optimized pepsin pre-treatment protocol [50] allowed the detection of ADAMTS-4 IR in extracellular protein depositions throughout the hippocampus of aged mice (lower and higher magnification). Scale bars: **A–C** = 500 µm; **E** = 200 µm.

To visualize the expression pattern of the Reelin proteases in the hippocampus, we performed an immunoperoxidase staining involving brain sections from 15 months old wild-type mice. In line with previous findings [48], [49], pyramidal cells represented the main source of tPA immunoreactivity (IR) (Fig. 6A). In addition, we also confirmed the pronounced tPA IR in the striatum lacunosum moleculare (slm, Fig. 6A) and mossy fibers [49] (data not shown). Similarly, ADAMTS-4 and -5 were prominently expressed by hippocampal pyramidal and Dentate Gyrus (DG) granular cells in wild-type mice (Fig. 6B,C), in line with the mRNA expression profile described in the Allen mouse brain atlas (http://www.brain-map.org). In particular, ADAMTS-4 IR was most prominent in the slm, but also found in striatum radiatum (sr) and the DG molecular layer (ml) (Fig. 6B). Highest ADAMTS-5 IR was detected in the sr and DG ml (Fig. 6C).

Since we have previously shown that Reelin aggregation was significantly increased in striatum oriens (so), sr and slm of transgenic 3xTg-AD mice as compared to wild-type controls [30], we analyzed the expression pattern of the Reelin proteases in these animals as well. Compared to controls, tPA IR was significantly increased in the so, slm, and DG ml of 15 month-old 3xTg-AD mice (Fig. 6A,D), while ADAMTS-5 IR was dramatically decreased in all layers of the hippocampus (Fig. 6C,D). At the same time, ADAMTS-4 IR seemed to be unchanged throughout the hippocampus (Fig. 6B,D) between genotypes. Note that at this age, the triple-transgenic mice show prominent Tau hyperphosphorylation, intracellular APP/Aβ accumulation, but no detectable Aβ plaque deposition in the dorsal hippocampus (Fig. S2A,B).

**Figure 7:**
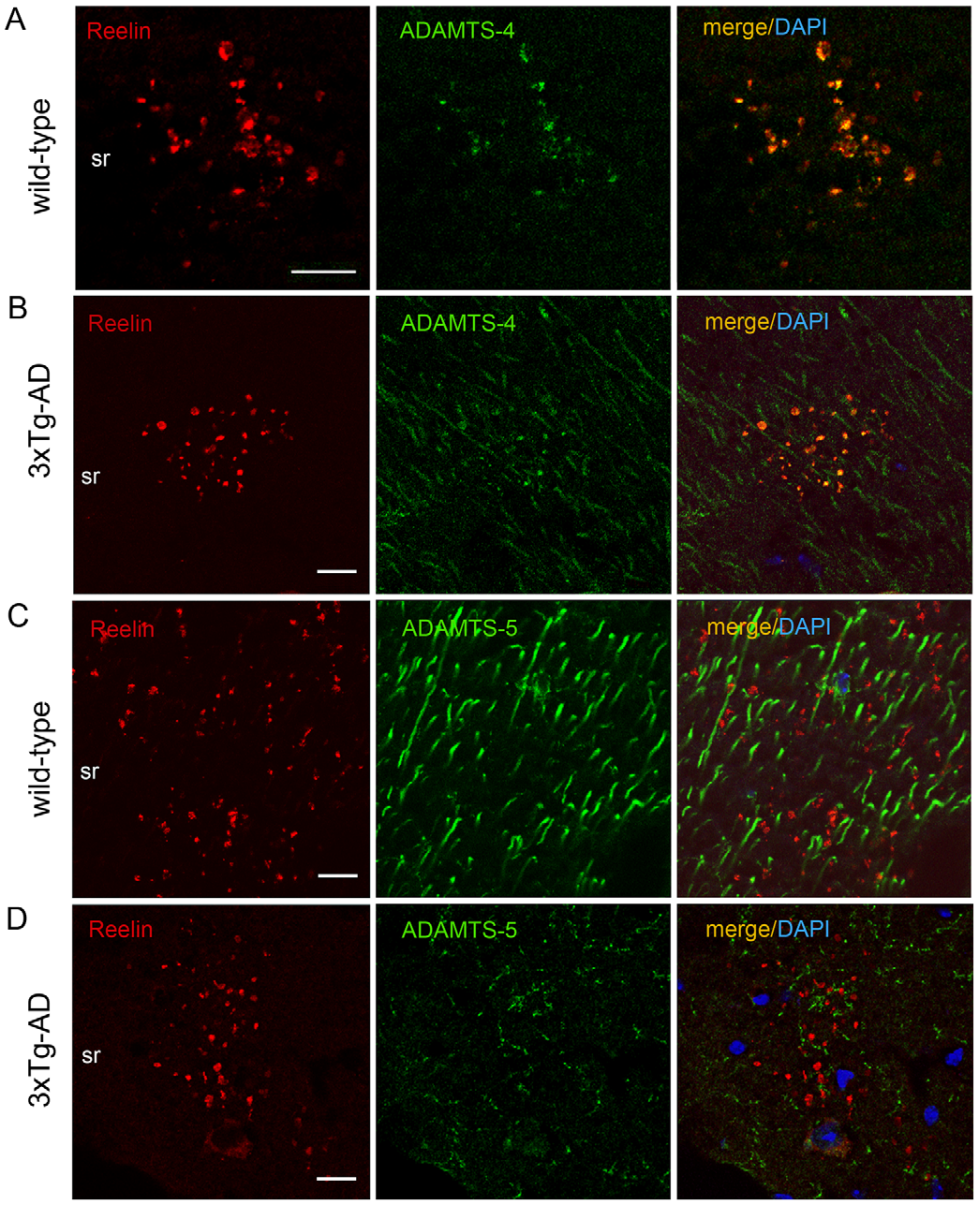
Co-localization of ADMTS-4 and Reelin in the *striatum radiatum* of aged animals. (**A–D**) Double-immunofluorescence staining using anti-Reelin (red) and anti- ADAMTS-4 and anti-ADAMTS-5 (green) antibodies on brain sections of 15 month-old non-transgenic and 3xTg-AD mice. Co-localization analysis revealed a selective overlap of ADAMTS-4 IR (**A,B**), but not ADAMTS-5 IR (**C,D**), with Reelin in extracellular deposits in the striatum radiatum (sr). DAPI (blue) counterstaining was used for labeling the cell nuclei. Scale bars: 20 µm.

The immunoperoxidase staining also revealed distinct ADAMTS-4 IR in extracellular deposits selectively in the so, sr, and slm of the hippocampus (Fig. 6E), brain areas where also Reelin aggregates [30]. Hence, we tested if the Reelin proteases could be detected within the Reelin aggregates. To this end, we utilized our recently described protocol for antigen retrieval and visualization of Reelin-positive amyloid-like plaques [50]. Co-immunofluorescence staining using anti-Reelin and anti-ADAMTS-4 or -5 antibodies confirmed a close association of Reelin with ADAMTS-4 in sr in both wild-type and 3xTg-AD mice (Fig. 7A,B), but not with ADAMTS-5 (Fig. 7C,D). No significant tPA signal was detected in sr (data not shown).

## Discussion

### Tissue Plasminogen Activator (tPA)

Here we demonstrate that tPA cleaves Reelin at its C-terminal cleavage site *in vitro* in both Reelin conditioned medium and HEK293 over-expressing cultures. Recently, it has been claimed that tPA - similarly to the urinary-type plasminogen activator (uPA) - could degrade Reelin in the plasma [36]. However, our data shows that the C-terminally cleaved Reelin fragment (NR6) is not a substrate for further degradation. Furthermore, in line with the observations that the C-terminal fragment of Reelin (R7-C) is required for proper protein folding [26] and full signaling activity [27], [28], we observed that a lack of the R7-C domain significantly affects the stability of the Reelin dimers and favors the formation of larger protein complexes. Interestingly, in addition to inhibiting tPA action on Reelin, neuroserpin and nexin also inhibited tPA-independent N-terminal cleavage, suggesting that these serpins may indirectly affect the maturation and/or activity of several ECM proteases. In addition, MMP-9-induced N-terminal cleavage of Reelin could be efficiently inhibited by the serine protease inhibitors SBTI and Aprotinin, which only marginally inhibited tPA and uPA [51], pointing to the possible interplay between MMP-9 and serpins in the regulation of Reelin cleavage through not yet identified serine protease(s).

### ADAMTS-4 and ADAMTS-5

In addition to tPA, we discovered that the metalloproteinases ADAMTS-4 and ADAMTS-5 cleave Reelin at both, the C- and N-terminal cleavage site. Interestingly, we also found that ADAMTS-5 is able to further degrade the N-terminal Reelin fragments. While the metalloproteinase inhibitor TIMP-4, shown to have a weak effect on ADAMTS-4 and -5 [42], did not inhibit Reelin cleavage in HEK293 cells, TIMP-3 and α2M - known to have a strong effect on ADAMTS-4 and -5 [42], [43] - inhibited Reelin cleavage in both cell free conditioned medium and HEK293 cell cultures. This is in accordance with the described action of piceatannol (PIC), a selective inhibitor of ADAMTS-4 and -5 [41]. Silencing experiments using shRNA against ADAMTS-4 showed a transient effect on Reelin cleavage in HeLa cell cultures. This may be due to either a “saturation” effect of the Reelin overexpression cell system, where high amounts of Reelin might mask slight changes induced by shRNA, or a concomitant upregulation of other proteases that are able to cleave Reelin. It will be interesting to test whether activation of the Reelin signaling cascade by the different Reelin fragments represent a putative autoregulatory feedback mechanism to control the level and activity of the Reelin cleaving proteinases. Although picetanol, a potent and specific inhibitor of ADAMTS-4 and -5, suppresses Reelin processing *in vitro*, shRNA against ADAMTS-5 yielded no inhibition in Reelin cleavage. Hence, we suggest that ADAMTS-4 and -5 may have both overlapping and compensatory functions in mediating Reelin proteolytic processing. Importantly, however, we showed that despite their high homology in the catalytic domains [47], not all members of the aggrecanase family are involved in Reelin cleavage.

### MMP-9

Based on our experimental data demonstrating that neither over-expression of MMP-9 in HEK293 cells nor the addition of recombinant MMP-9 into pre-boiled FL-Reelin medium was sufficient to induce Reelin cleavage, we conclude that MMP-9 is only indirectly involved in the proteolytic processing of Reelin, likely by regulating the activity of ADAMTS-4. Interestingly, our investigations of the effect of the different proteinase inhibitors indicates that in addition to α2M, a potent inhibitor of ADAMTS-4 and -5, also a serine protease inhibitor mix (SBTI/Apro) inhibited Reelin N-terminal cleavage, pointing to the involvement of additional serine proteases in the regulation of ADAMTS-4 and/or -5 activity. However, with the present set of data we cannot completely rule out the possibility that MMP-9 can directly cleave Reelin at the C-terminal site. Also, it is possible that HEK293 cells may compensate for the overexpression of MMP-9 by the induction of MMP-9 inhibitors.

### Reelin proteases *in vivo*

Importantly, while biochemical analysis confirmed the presence of all identified Reelin proteases in the brain, immunohistochemical analyses of the adult murine hippocampus revealed that the expression patterns of these proteases largely overlap with that of Reelin [30].

Reelin itself accumulates in amyloid-like deposits in the brain, representing a common feature of normal aging in several species [30]. This phenomenon is significantly accelerated upon prenatal infection in wild-type mice [30], [52] and strongly aggravated in transgenic AD mouse models [30], [53]. I*n vitro* evidence suggests that this aggregation may be linked to dysregulated Reelin cleavage, since the proteolytic processing of full-length Reelin not only affects its signaling properties via compulsory Reelin dimers [19], [20], [27], [28], but also induces the formation of complexes/aggregates of larger molecular mass than the dimers [20], [29] and (Fig. 1F). Unexpectedly, our semi- quantitative analysis on the hippocampus lysates from young (4 weeks) and aged (16 months) wild-type mice did not reveal any changes in Reelin proteolytical processing or a change in protein levels of the Reelin proteases across aging – despite the presence of numerous Reelin aggregates. However, in contrast to young animals, a smaller N-terminal Reelin fragment with a molecular weight of approximately 60 kDa was invariantly present in significant amounts in the hippocampus of old wild-type mice. Hence we conclude that the age-dependent decrease in proper protein degradation [54] may also affect Reelin, which in turn may lead to the intraneuronal accumulation of aggregation-prone N-terminal fragments. This hypothesis is in line with our recent 3D immuno-electron microscopical analyses that provided the first evidence that Reelin deposits in the hippocampus originate intracellularly and are extruded into the ECM, where they can be cleared by microglia and astrocytes [52]. The presence of ADAMTS-4 in these deposits/aggregates suggests, therefore, that ADAMTS-4-dependent cleavage occurs after Reelin internalization, probably to terminate Reelin-mediated signaling. This is also in agreement with recent findings demonstrating that in HEK293 cells N-terminal Reelin fragments are produced after receptor-mediated endocytosis [55].

Based on our previous findings of a significantly increase in Reelin aggregates in the hippocampus of 15 month-old 3xTg-AD mice as compared to age-matched controls [30], we performed a complementary immunohistochemical analysis of the identified proteases in these mice. We observed significant changes in the levels of tPA and ADAMTS-5, but not ADAMTS-4, between genotypes. While tPA levels increased in the striatum lacunosum moleculare (slm) in AD-mice as compared to controls, ADAMTS-5 levels dramatically decreased throughout all layers of the hippocampus of the transgenic mice. Since ADAMTS-5 is most prominently expressed in the dendrites of the pyramidal neurons of the hippocampus and its protein levels are dramatically decreased in old 3xTg-AD mice, it is plausible that the acceleration of the Reelin aggregation in striatum radiatum of AD mice is, at least in part, due to the decreased Reelin degradation by ADAMTS-5. Finally, since extracellular Reelin immunoreactivity (IR) is very prominent in the slm, we suggest that tPA-induced Reelin cleavage may modulate the synaptic function in the CA1 region. Although further investigations are required to clarify the potential regulatory role of tPA on Reelin-mediated signaling, a plausible effect of an increased C-terminal cleavage is expected to include a concomitant decrease in the induction of the downstream signaling pathways in the CA1 pyramidal neurons [27], [28] including an increase in hyperphosphorylation of Tau [16], [17]. This hypothesis is in accordance with our recent *in vivo* data demonstrating that a genetic reduction of Reelin, and thus reduction of its signaling, leads to formation of neurofibriallary-like tangles (NFTs) in AD-mice in the absence of human *tau* transgene overexpression [53].

### Conclusion

Here we demonstrate that the serine protease tPA as well as metalloproteinases ADAMTS-4 and -5, proteolytically process Reelin, pointing to a complex interplay between multiple proteinases in modulating Reelin cleavage (Fig. 4F). While tPA cleaves Reelin only at its C-terminus, ADAMTS-4 and -5 cut at both, the N- and C-cleavage site. In addition, ADAMTS-5 is able to further degrade the N-terminal fragment. Despite common roles in cartilage matrix degradation, we show that not all members of the aggrecanase family cleave Reelin, pointing to the specificity of Reelin proteolytic processing by ADAMTS-4 and -5. Furthermore, enzymatic action of tPA on Reelin could be inhibited by serpin E1 (nexin) or serpin I1 (neuroserpin), and the actions of ADAMST-4 and ADAMTS-5 by the endogenous inhibitors of matrix metalloproteases; TIMP-1/-3 as well as α2M. Moreover, we observed that serpins and MMP-9, in addition to modulation of tPA and ADAMTS-4 respectively, also have a tPA- and ADAMTS-independent role in the regulation of Reelin processing. This may involve also other, yet unidentified, proteases (Fig. 4F). Importantly, tPA, ADAMTS-4 and -5 are expressed in the brain and their expression patterns in the hippocampus largely overlaps with that of Reelin. While no significant changes in protein levels of these proteases as well as in Reelin processing were detected during normal aging, the protein levels of tPA and ADAMTS-5 drastically changed in AD-mice compared to non-transgenic controls. Further studies, including protease knock-out mice, will be required to better understand the regulation these proteases and their impact on Reelin-mediated signaling during normal and pathological forms of aging.

## Materials and Methods

### Cells

HEK293 and HeLa cells were obtained from American Type Culture Collection (ATCC, Manassas, VA, USA). The cells were grown as a monolayer in DMEM (Invitrogen, Carlsbad, CA, USA) containing 5% Fetal Calf Serum (Gibco Invitrogen).

### Animals

All experimental procedures were approved by the local authorities of the Cantonal Veterinary Office in Zurich. Animals were housed in groups of 3-4 in an optimized in-house hygiene area (OHB, University of Zurich Irchel, Switzerland) under 12-h day-night cycle and *ad libitum* food and water. Kainate injections were performed as described previously [56]. Transgenic AD-mice (3xTg-AD; encoding APPSwe, and TauP301L on a homozygous PS1M146V knock-in background) [33] and the non-transgenic mice with the same genetic background (129/C57Bl6) were obtained from Dr. Frank LaFerla (University of California Irvine).

### Reagents

The following reagents were used: Furin Inhibitor I (FI-I; Merck, Zug, Switzerland), Bis(sulfo-succinimidyl) suberate sodium salt (BS3; Thermo Scientific Pierce, Rockford, IL, USA; BS3 crosslinking of FL- and C-cleaved Reelin was performed as described previously) [20], Trypsin inhibitor (SBTI; Sigma-Aldrich, St. Louis, MO, USA), Aprotinin (Apro; Roche Applied Science, Mannheim, Germany), 1,10-Phenanthroline (PO; Sigma-Aldrich), Epigallocatechin gallate (EGCG; Sigma-Aldrich), Epicatechingallate (ECG; Sigma-Aldrich), Piceatannol (PIC; Sigma-Aldrich), DMSO (Sigma-Aldrich).

### Plasmids and Recombinant Proteins

All expression plasmids used in this study contained human cDNA sequences and were obtained from OriGene Technologies, Rockville, MD. For expression studies involving ADAMTS-4 and ADAMTS-5, human TrueORF plasmids (OriGene) were utilized. shRNA against ADAMTS-4,-5 and tPA were obtained from OriGene. The following recombinant human proteins were used: ADAMTS-1 and -4 (from N terminus of the catalytic domain to the beginning of the spacer region; Phe253-Ala734 and Phe213-Cys683, respectively), ADAMTS-5 (from N terminus of the catalytic domain to the C terminus of the TSP1 domain; Ser262-Pro622), TIMP-3, alpha-2-macro-globulin, serpineE1/PAI-1 (all from R&D Systems, Minneapolis, MN, USA), MMP-9 (PF140 active, Calbiochem, Schwalbach, Germany), and tPA Tissue Plasminogen Activator (activated, Abcam, Cambridge, UK). All experiments involving recombinant proteins were performed at 37°C, and the time after the addition of the proteins into FL-Reelin (uncleaved) medium was measured between 0 and 24 h.

### Transfections

HEK293 and HeLA cells were transfected with LipofectamineTM 2000 transfection reagent (Invitrogen) according to manufacturer instructions. Medium changes were performed at 12 h post-transfection (changed to DMEM including 5% FCS), and subsequently at 24 h post-transfection (changed to AIM V® Serum Free Medium Invitrogen, with or without various inhibitors). The amount of transfected DNA was always kept constant by addition of an empty vector cDNA. Sample (AIM V medium) was collected after 12-24 h incubation of transfected HEK293 cells at 37°C.

### Reelin conditioned media

To obtain uncleaved full-length Reelin, we transfected HeLa cells as described above with the Reelin-pCRL plasmid [57], kindly provided by Dr. Tom Curran, and 24 h after transfection the medium was exchanged with AIM V containing 100 µM FI-I dissolved in DMSO (final concentration in medium 2%). The medium was collected after 12 h, aliquoted and stored at -80°C until further usage. The time of incubation is indicated in all figures presenting data which involve this type of prepared medium with recombinant proteins or control protease buffer.

### Immunoblotting (IB)

Sample preparation and Western Blotting protocol was carried out as described before [55], with the following changes for the Reelin Western Blots: The samples were prepared using NuPAGE®LDS sample buffer and DTT reducing reagent (Invitrogen). Samples were heated to 70°C for maximum 5 min, separated on 3–8% gradient Tris-Acetate gels (Invitrogen), and the proteins were blotted on PVDF membranes (Invitrogen). After transfer, membranes were blocked for 30 min with 1% solution of Western Blocking Reagent (Roche Applied Science). Before and after secondary antibody incubation, the membranes were washed for 1 h (6×10 min) in TBST washing buffer.

### Immunohistochemistry (IHC)

Tissue preparation, IHC protocol, and microscopy was performed as described previously [30], [50], [55]. Intensity of the immunoperoxidase signal (pixel brightness) and co-localization of the fluorescent signals (pixel overlap) were analyzed utilizing ImageJ software (NIH, Bethesda, MD).

### Antibodies

Mouse anti-Reelin (clone G10, Millipore, Billerica, MA, USA, IB/IHC 1∶1000), mouse anti-Reelin (clone 142, Millipore, IB 1∶1000), goat anti-tPA (387, American Diagnostica Inc., Stanford, CT, USA, IHC 1∶250) [58], [59], rabbit anti-tPA (H-90, Santa Cruz Biotechnology, Santa Cruz, CA, USA, IB 1∶500) [60], mouse anti-ADAMTS-1 (3E4C6B4, Santa Cruz Biotechnology, IB 1∶500), rabbit anti-ADAMTS-4 (PA1-1749A, Thermo Scientific, IB 1∶1000, IHC 1∶500-1000), rabbit anti-ADAMTS-5 (ab41037, Abcam, IB/IHC 1∶1000) [61], rabbit anti-ADAMTS-5 (PA1-1751A, Thermo Scientific, IB/IHC 1∶1000) [62], rabbit anti-MMP-9 (ab38898, Abcam, IB 1∶1000), rabbit anti-Aβ1-40/42 (AB5075, Millipore, IHC 1∶2000), and rabbit anti-pTauT205 (ab4811, Abcam, IHC 1∶1000), mouse anti-Actin (MAB1522, Millipore, IB 1∶40′000).

### Statistics

All comparisons were performed with GraphPad Prism (GraphPad Software, San Diego, CA, USA), utilizing unpaired t-test with Welch's correction. Statistical significance was set at p<0.05.

## Supporting Information

Table S1 **Reelin protease candidates.** Adapted from Hatada and colleagues [35]. Analysis of the microarray data [35] yielded 19 serine proteases, 20 metalloproteinases, 4 convertases, and 10 proteinase inhibitors being up-regulated upon retinoic-acid induced differentiation of embryonic P19 tetracarcinoma cells into neurons.
(TIF)

Figure S1 **Supplementary immunoblot data.** (**A–E**) Anti-Reelin (G10, N-terminal antibody) immunoblots (IB). For all panels: hours (h) represent incubation time; short vertical lines at the bottom of some blots denote that the last lanes, from the same blot, were joined for visual display. All IB blots are representatives of three independent experiments. **(A–C)** Reelin-expressing HEK293 cells were incubated with different concentrations of 1,10-Phenanthroline (PO), Epigallocatechin (EGCG), Epicatechingallate (ECG), and Piceatannol (PIC) for 12 h. High concentrations of the inhibitors prevent both, the N- and C-terminal cleavage. Lower concentrations of catechin EGCG blocks only N-terminal but not C-terminal cleavage of Reelin. Similarly, lower concentrations of catechin ECG had a more prominent inhibitory effect on N-terminal cleavage. (**C**) Test of the effect of DMSO (solvent for piceatannol) on HEK293 cells, serving as internal control (con). **(D)** Incubation of the trypsin inhibitors SBTI and Apro with active ADAMTS-4 (10 ng/µl), did not inhibit Reelin cleavage. (**E**) Immunoblot showing no Reelin degradation/fragmentation after the FL-Reelin medium was heated at 80°C (lane 2) and 90°C (lane 3) for 10 minutes. Blot has been overexposed to better visualize potential weak degradation. As a result of the 1 h overexposure, the pre-stained molecular weight marker included in lane 1 shows strong non-specific signals that are absent after shorter exposure times. (**F**) Immunoblots using anti-Reelin G10, N-terminal antibody (lanes 1,2) and anti-Reelin 142, N-terminal antibody (lanes 3,4). Lanes represent hippocampus homogenates from young (lanes 1,3) and old (lanes 2,4) wild-type mice. Blots are overexposed to visualize the degradation bands of N-terminal fragments of Reelin. Both antibodies recognize degradation bands at ∼130 and ∼100 kDa (asterisk, as described in Fig. 4A,B) and a band at ∼60 kDa (two asterisks) that selectively appears in the old animals (as described in Fig. 5D,E). Note that a strong band at ∼70 kDa could be observed only with the anti-Reelin 142 antibody. **(G)** Expression of ADAMTS-1 did not increase Reelin cleavage in Reelin expressing HEK293 cells (left), although synthesis of ADAMTS-1 protein was confirmed using anti-ADAMTS-1 antibody (right).
(TIF)

Figure S2 **Supplementary immunohistochemistry data.** **(A,B)** Immunoperoxidase staining using Aβ_1−40/42_ (**A**) and phospho-Tau (pTau^T205^, **B**) antibodies on brain tissue obtained from 15 month-old non-transgenic and 3xTg-AD mice. At this stage, no plaque deposition could be detected in the dorsal hippocampus (**A**), but strong transgene-induced Tau phosphorylation was observed in the pyramidal neurons of the CA1 region (**B**). (**C**) To test ADAMTS-4 antibody specificity for immunohistochemistry, we performed immunoperoxidase staining on brain tissue obtained from 3 month-old wild-type control (left) and kainate-treated (0.2 µg kainic acid, injected in a volume of 70 nl into the dorsal CA1 region [56] mice (right, only contralateral hippocampus is shown). As expected, from the *in situ* hybridisation data [63], kainate injection elevated the expression of ADAMTS-4 in the pyramidal and granular cells of the hippocampus. (**D**) To test ADAMTS-5 antibodies specificity for immunohistochemistry, we performed immunoperoxidase staining using ADAMTS-5 (PA1-1751A) antibody on brain tissue obtained from 15 month-old wild-type mice. PA1-1751A antibody revealed a highly similar expression pattern to that of ab41037 (compare left panel with left pannel in Fig. 6C). Although the intensity of the signal was weaker in the dendritic fields of CA1 with PA1-1751A than with ab41037, the PA1-1751A antibody also revealed a strong decrease in ADAMTS-5 protein levels in the hippocampus of aged 3xTg-AD-mice as compared to non-transgenic controls (right). Scale bars: **A–B** = 500 µm.
(TIF)
