# Supplementary figures and images for: Regulated Proteolytic Processing of Reelin through Interplay of Tissue Plasminogen Activator (tPA), ADAMTS-4, ADAMTS-5, and Their Modulators
Source: PLoS One. 2012 Oct 17;7(10):e47793. doi: 10.1371/journal.pone.0047793 (PMC3474754; doi:10.1371/journal.pone.0047793)

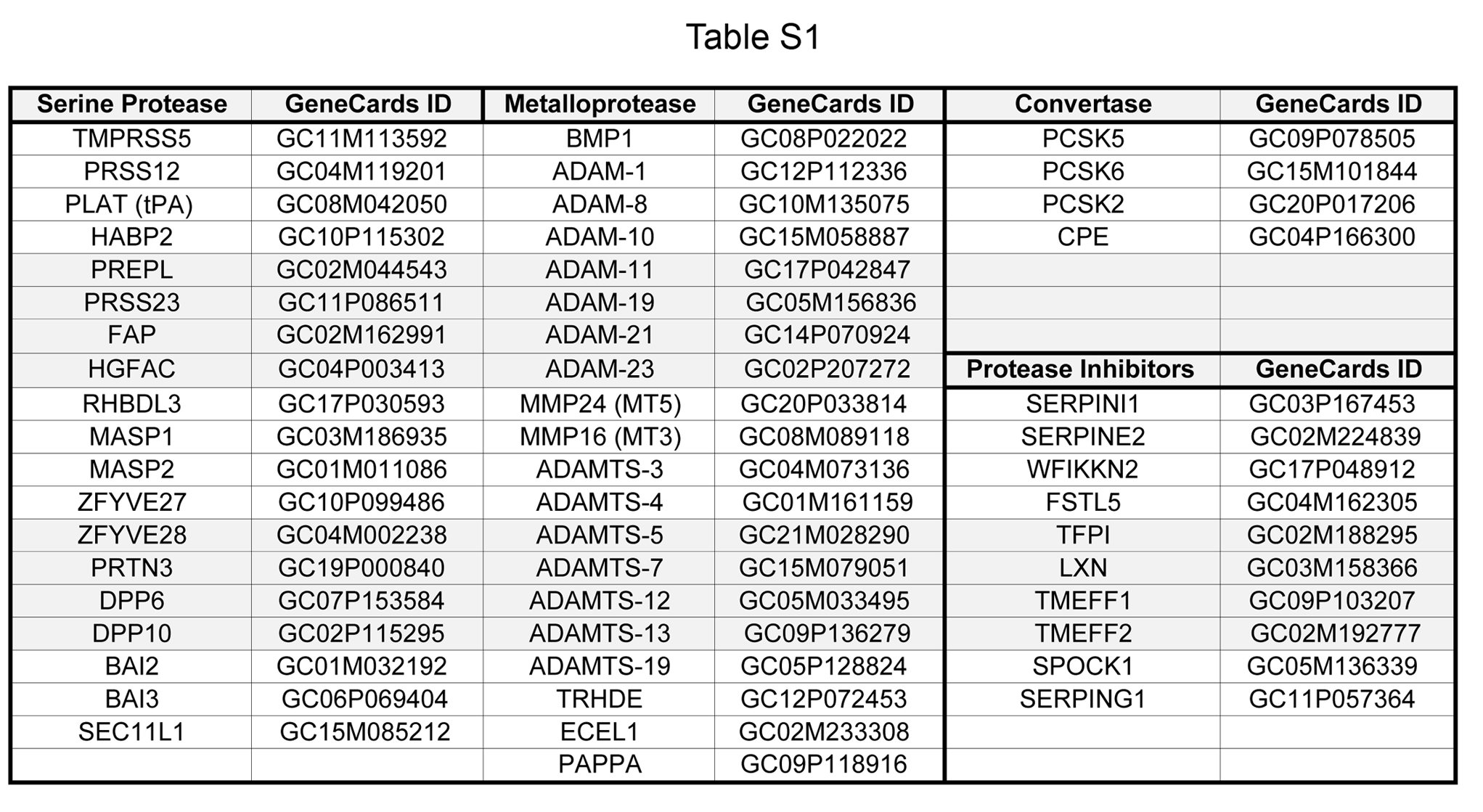

Supplement: Table S1 — Reelin protease candidates. Adapted from Hatada and colleagues [35]. Analysis of the microarray data [35] yielded 19 serine proteases, 20 metalloproteinases, 4 convertases, and 10 proteinase inhibitors being up-regulated upon retinoic-acid induced differentiation of embryonic P19 tetracarcinoma cells into neurons. (TIF) [file pone.0047793.s001.tif]

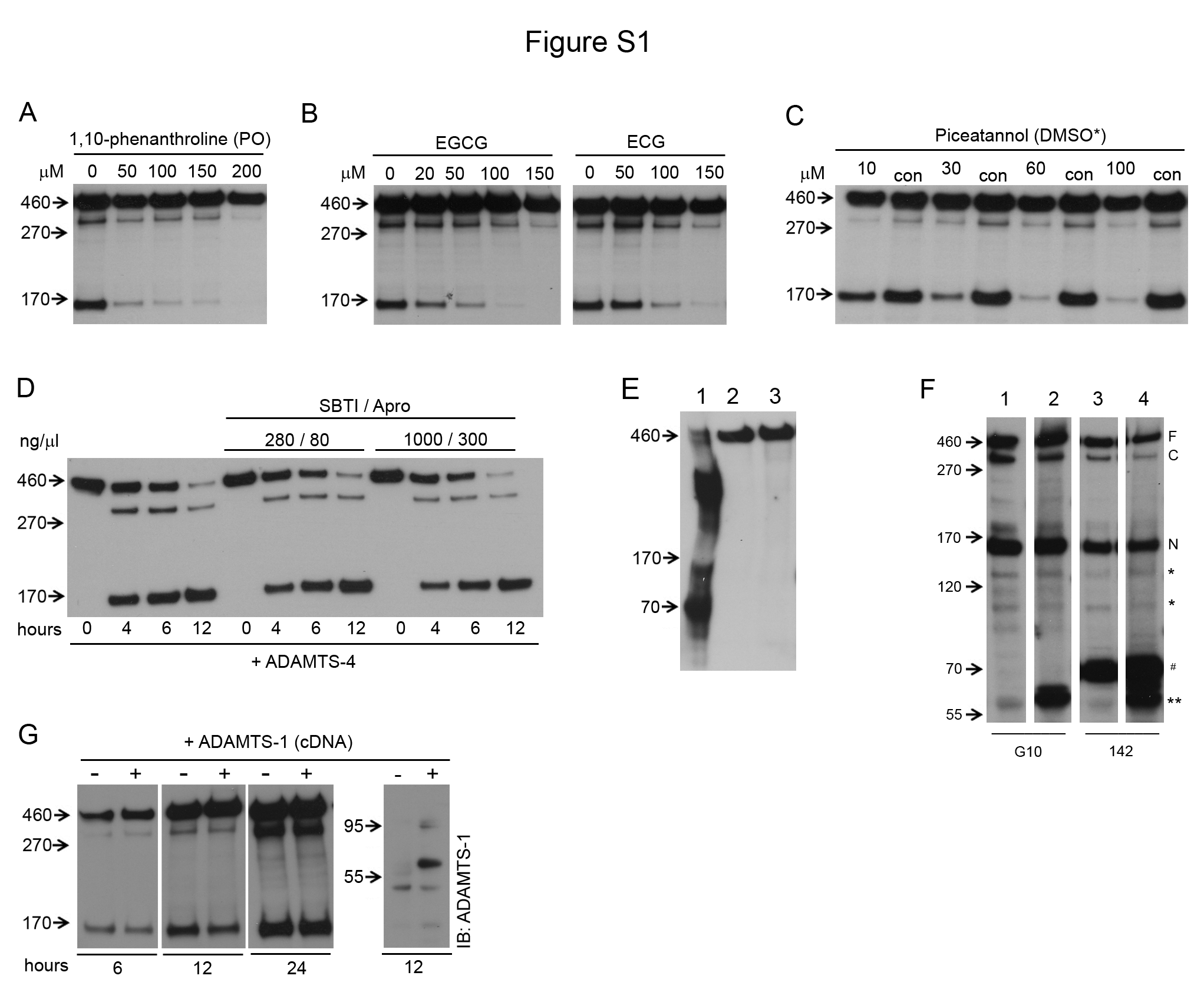

Supplement: Figure S1 — Supplementary immunoblot data. (A–E) Anti-Reelin (G10, N-terminal antibody) immunoblots (IB). For all panels: hours (h) represent incubation time; short vertical lines at the bottom of some blots denote that the last lanes, from the same blot, were joined for visual display. All IB blots are representatives of three independent experiments. (A–C) Reelin-expressing HEK293 cells were incubated with different concentrations of 1,10-Phenanthroline (PO), Epigallocatechin (EGCG), Epicatechingallate (ECG), and Piceatannol (PIC) for 12 h. High concentrations of the inhibitors prevent both, the N- and C-terminal cleavage. Lower concentrations of catechin EGCG blocks only N-terminal but not C-terminal cleavage of Reelin. Similarly, lower concentrations of catechin ECG had a more prominent inhibitory effect on N-terminal cleavage. (C) Test of the effect of DMSO (solvent for piceatannol) on HEK293 cells, serving as internal control (con). (D) Incubation of the trypsin inhibitors SBTI and Apro with active ADAMTS-4 (10 ng/µl), did not inhibit Reelin cleavage. (E) Immunoblot showing no Reelin degradation/fragmentation after the FL-Reelin medium was heated at 80°C (lane 2) and 90°C (lane 3) for 10 minutes. Blot has been overexposed to better visualize potential weak degradation. As a result of the 1 h overexposure, the pre-stained molecular weight marker included in lane 1 shows strong non-specific signals that are absent after shorter exposure times. (F) Immunoblots using anti-Reelin G10, N-terminal antibody (lanes 1,2) and anti-Reelin 142, N-terminal antibody (lanes 3,4). Lanes represent hippocampus homogenates from young (lanes 1,3) and old (lanes 2,4) wild-type mice. Blots are overexposed to visualize the degradation bands of N-terminal fragments of Reelin. Both antibodies recognize degradation bands at ∼130 and ∼100 kDa (asterisk, as described in Fig. 4A,B) and a band at ∼60 kDa (two asterisks) that selectively appears in the old animals (as described in Fig. 5D,E). Note that [file pone.0047793.s002.tif]

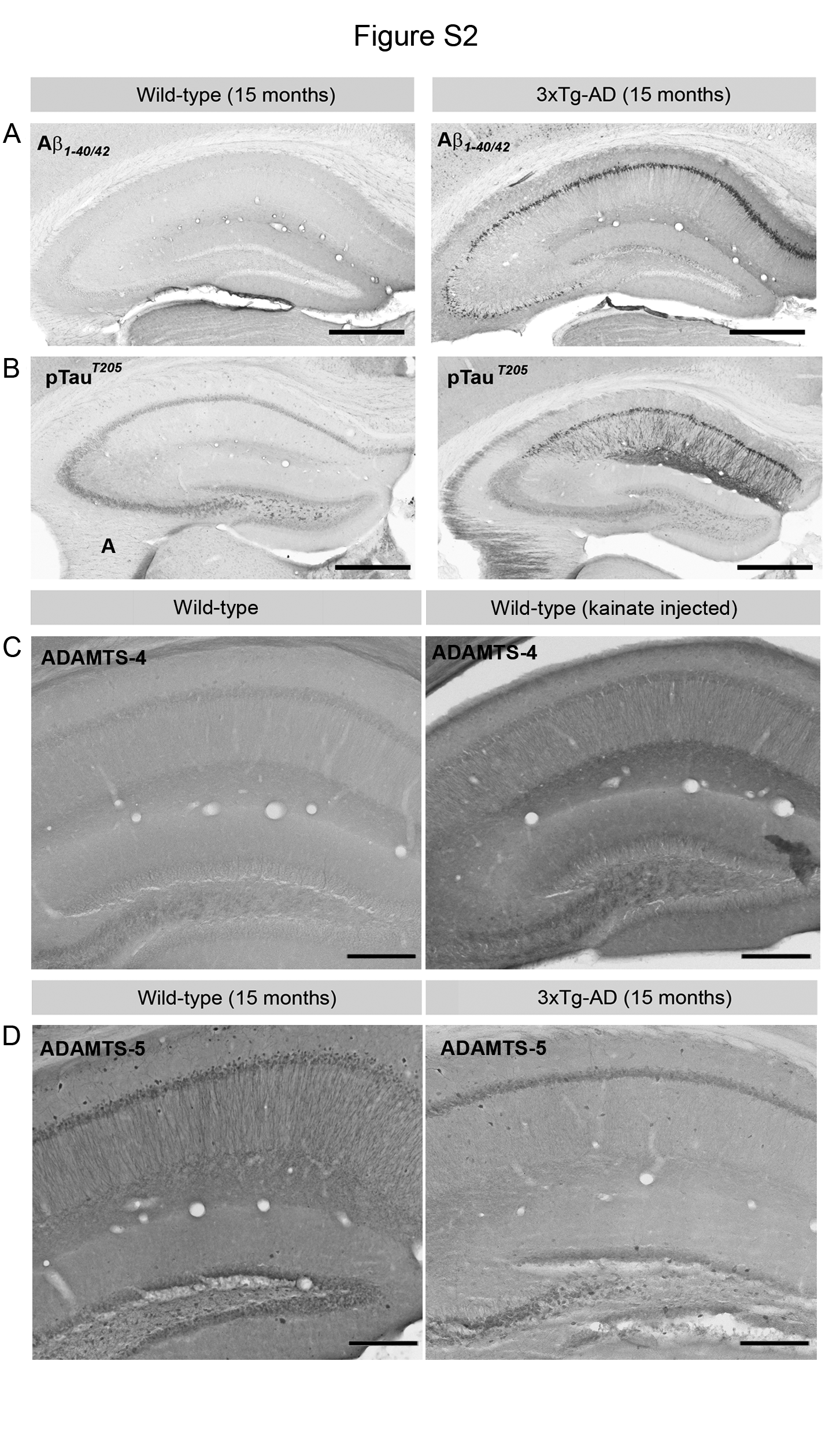

Supplement: Figure S2 — Supplementary immunohistochemistry data. (A,B) Immunoperoxidase staining using Aβ1−40/42 (A) and phospho-Tau (pTauT205, B) antibodies on brain tissue obtained from 15 month-old non-transgenic and 3xTg-AD mice. At this stage, no plaque deposition could be detected in the dorsal hippocampus (A), but strong transgene-induced Tau phosphorylation was observed in the pyramidal neurons of the CA1 region (B). (C) To test ADAMTS-4 antibody specificity for immunohistochemistry, we performed immunoperoxidase staining on brain tissue obtained from 3 month-old wild-type control (left) and kainate-treated (0.2 µg kainic acid, injected in a volume of 70 nl into the dorsal CA1 region [56] mice (right, only contralateral hippocampus is shown). As expected, from the in situ hybridisation data [63], kainate injection elevated the expression of ADAMTS-4 in the pyramidal and granular cells of the hippocampus. (D) To test ADAMTS-5 antibodies specificity for immunohistochemistry, we performed immunoperoxidase staining using ADAMTS-5 (PA1-1751A) antibody on brain tissue obtained from 15 month-old wild-type mice. PA1-1751A antibody revealed a highly similar expression pattern to that of ab41037 (compare left panel with left pannel in Fig. 6C). Although the intensity of the signal was weaker in the dendritic fields of CA1 with PA1-1751A than with ab41037, the PA1-1751A antibody also revealed a strong decrease in ADAMTS-5 protein levels in the hippocampus of aged 3xTg-AD-mice as compared to non-transgenic controls (right). Scale bars: A–B = 500 µm. (TIF) [file pone.0047793.s003.tif]
